# The genetic prehistory of domesticated cattle from their origin to the spread across Europe

**DOI:** 10.1186/s12863-015-0203-2

**Published:** 2015-05-28

**Authors:** Amelie Scheu, Adam Powell, Ruth Bollongino, Jean-Denis Vigne, Anne Tresset, Canan Çakırlar, Norbert Benecke, Joachim Burger

**Affiliations:** Johannes Gutenberg-University Mainz, Institute of Anthropology, Palaeogenetics Group, 55099 Mainz, Germany; German Archaeological Institute, Scientific Department, Im Dol 2-6, Haus 2, 14195 Berlin, Germany; Muséum National d’Histoire Naturelle, UMR7209, ”Archéozoologie, archéobotanique: sociétés, pratiques et environnements”, InEE, Département d’Ecologie et Gestion de la Biodiversité, CP 56, 55 rue Buffon, 75005, Paris, Cedex 05 France; University of Groningen, Institute of Archaeology, Poststraat 6, NL-9712 ER, Groningen, Netherlands

## Abstract

**Background:**

Cattle domestication started in the 9^th^ millennium BC in Southwest Asia. Domesticated cattle were then introduced into Europe during the Neolithic transition. However, the scarcity of palaeogenetic data from the first European domesticated cattle still inhibits the accurate reconstruction of their early demography. In this study, mitochondrial DNA from 193 ancient and 597 modern domesticated cattle (*Bos taurus*) from sites across Europe, Western Anatolia and Iran were analysed to provide insight into the Neolithic dispersal process and the role of the local European aurochs population during cattle domestication.

**Results:**

Using descriptive summary statistics and serial coalescent simulations paired with approximate Bayesian computation we find: (i) decreasing genetic diversity in a southeast to northwest direction, (ii) strong correlation of genetic and geographical distances, iii) an estimated effective size of the Near Eastern female founder population of 81, iv) that the expansion of cattle from the Near East and Anatolia into Europe does not appear to constitute a significant bottleneck, and that v) there is evidence for gene-flow between the Near Eastern/Anatolian and European cattle populations in the early phases of the European Neolithic, but that it is restricted after 5,000 BCE.

**Conclusions:**

The most plausible scenario to explain these results is a single and regionally restricted domestication process of cattle in the Near East with subsequent migration into Europe during the Neolithic transition without significant maternal interbreeding with the endogenous wild stock. Evidence for gene-flow between cattle populations from Southwestern Asia and Europe during the earlier phases of the European Neolithic points towards intercontinental trade connections between Neolithic farmers.

**Electronic supplementary material:**

The online version of this article (doi:10.1186/s12863-015-0203-2) contains supplementary material, which is available to authorized users.

## Background

The transition from foraging to producing economies, also called Neolithisation, was a major turning-point in human prehistory. The process of Neolithisation started in a region spanning from the Zagros Mountains to Central Anatolia and from Palestine to the plains beyond the East Taurus Mountains [1,2]. It was characterized by the successive appearance of sedentism (12^th^-10^th^ millennia BCE), plant cultivation (mid-10^th^ millennium), animal husbandry (mid-9^th^ millennium) and pottery (early 7^th^ millennium) [3,4]. Elements of the Neolithic lifestyle expanded into Western Anatolia in the early 7^th^ millennium [5-7], while the earliest signs for Neolithic settlements on the European continent are found in present-day Greece around 6,400 BCE [8]. The subsequent Neolithic spread across the rest of Europe followed at least two main routes: One leading across Southeastern Europe, and the second via the Western Mediterranean [9-11]. The extent to which this expansion of a new culture and economy was driven by the migration of people has been debated for decades [12-17]. An early study on human ancient DNA emphasized the role of inward migration at the beginning of the Neolithic period in Central Europe [18], a view that is supported by more recent palaeogenomic studies [19,20].

As animal husbandry was an important part of the foundation of the new agricultural lifestyle, remains of domesticated animals can serve as a good proxy for the Neolithic spatial expansion and the presence and activity of farmers in newly populated areas [21]. In recent years, genetic and palaeogenetic studies have increasingly converged on a Southwest Asian origin for the four Neolithic domesticated animals: cattle, sheep, goats, and pigs [22]. For Near Eastern taurine cattle (*Bos taurus*), a recent coalescent-based analysis using ancient Iranian samples suggested a severe Near Eastern domestication bottleneck, with an estimated effective size of just 80 female founders [23]. However, comprehensive data sets of ancient cattle DNA from other areas are so far restricted to Central and Western Europe, for example from Bollongino *et al.* [24]. Thus, detailed and continent-wide evaluation of the early spatiotemporal demography of *Bos taurus* has so far been hindered by the lack of data from the key bridging areas of the Neolithic, namely Anatolia, the Balkans, and the Western Mediterranean.

In this study we greatly extend a previous coalescent-based demographic model, based on 15 ancient Iranian and 27 modern Near Eastern and Anatolian cattle mitochondrial DNA (mtDNA) sequences, in terms of sample size, and geographic and temporal range [23]. To be able to investigate the early population history and migratory patterns of taurine cattle in detail, the present model is now conditioned on a larger ancient (*n* = 193, including the Iranian samples) and modern (*n* = 597) mtDNA dataset that widely covers the area of the Neolithic westwards expansion from the 7th millennium BCE onwards. The focus of the study is on the time period when cattle were first introduced to Europe, thereby allowing us to address the following questions: i) Is the scenario of a single and severe domestication bottleneck in the Near East still supported when adding the much larger dataset from western Anatolia and Europe? ii) Did cattle reach Europe in a single dispersal process or is there evidence for multiple introductions or continuous gene-flow between regions? iii) How much of the genetic diversity from the Near East was introduced to the European continent? iv) Did the spread of cattle coincide with the spread of the Neolithic culture? and v) Are there any signs of admixture with female aurochs along the expansion route of domestic cattle?

## Methods

### Material

150 samples of prehistoric domestic cattle from 24 archaeological sites were taken to analyse a 434 bp long mitochondrial d-loop fragment (for detailed information on the archaeological sites and sample age see Additional file 1. The majority of investigated individuals (113) come from Western Anatolia and Southeastern Europe, i.e. a region defined as an “interim zone” [5], pointing to its bridging position between the “Neolithic core zone” and its European fringe.

A further 22 samples from Southern France and Southern Italy represent the first domesticated cattle to reach Europe on the “Mediterranean route” of the Neolithic expansion.

Additionally, new prehistoric samples from Germany (4), Northern and Western France (11) and Syria (1), plus 80 previously published sequences mainly from Central and Western Europe and Iran were used for population genetic analyses ([23-28] and GenBank: KC172647 - KC172649). A total of 597 modern d-loop sequences of 240 bp length were collected from previously published studies [29,30]. They each provide representative sets of sequences that match the European, Anatolian and Near Eastern study area of the present paper, thereby also covering areas which are underrepresented in the aDNA dataset, e.g. Italy and the Iberian Peninsula. For a complete list of GenBank accession numbers of previously published sequences see Additional file 2.

### Ancient DNA work and sequencing

All samples were processed in the ancient DNA facilities at the Institute of Anthropology, Mainz University (Germany), under strict rules for contamination prevention as described in Bramanti *et al.* [18]. Those include strict separation of pre-PCR and post-PCR labs, protective clothes, regular cleaning of surfaces and equipment with detergent and bleach, and UV-irradiation of rooms, laboratory hoods, and equipment. Bone samples were UV-irradiated for 45 min from two sides. The surface was mechanically removed using a sandblaster (P-G 400, Harnisch & Rieth) or rotary saw (Electer Emax IH-300, MAFRA). Bone cubes of about 0.3 cm side length were again UV-irradiated for 45 min from two sides. Samples were pulverized using a mixer mill (MM200, Retsch). Generally, aliquots of 0.5 g bone powder were incubated on a rocking shaker at 37°C in a decalcification and digestion solution containing 2.5 ml EDTA (0.5 M, pH8; Ambion®/Applied Biosystems), 250 μl N-Laurylsarcosine (0.5 %; Merck) and 30 μl Proteinase K (18 U; Roche). DNA extraction was performed using phenol-chloroform-isoamylalcohol (25:24:1; Roth). DNA was washed and concentrated using 50 kDa Centricons or 50 kDa 15 ml Amicons (Millipore). At least two independent extractions per sample were performed. Extraction blank controls were processed during each extraction. Additionally, the cleanness of the grinding jars was tested by extracting hydroxylapatite that was pulverized under the same conditions as the bone samples.

Amplification of 434 bp of the HVSI (positions 15914–9 according to reference sequence V00654) was generally conducted using a PCR primer set consisting of 6 primer pairs as in Bollongino *et al.* [23] (BosU1/L1-BosU6/L6) with slight modifications.

PCR reactions were usually performed with 2.5 U AmpliTaq Gold® (Applied Biosystems), 1x PCR Gold Buffer (Applied Biosystems), 2 mM MgCl_2_ (Applied Biosystems), 0.2 mM dNTP’s (Quiagen), 0.4 μg/μl BSA (Roche), and 0.2 μM primer (Biospring) and HPLC-H_2_0 (Acros Organics). Initial activation at 90°C for 6 min was followed by 50 cycles of denaturation (40 sec at 94°C), annealing (40 sec at 52-60°C), and elongation (40 sec at 72°C) in a Mastercycler gradient (Eppendorf). Blank controls were processed during each PCR. At least three independent PCRs from two different extracts were performed. Samples were sequenced on an ABI PRISM™ 3130 Genetic Analyzer (Applied Biosystems) using POP-6™ polymer (Applied Biosystems).

Sequences were analysed using the programs SeqMan™ and MegAlign™ (DNASTAR Lasergene® 7.1 and 8). At least three sequences obtained from independent PCRs from two independent DNA extractions per sample per primer pair were usually used to create a majority rule consensus sequence. For further details on ancient DNA work and sequencing including deviations from the general laboratory procedure described see Additional file 3.

### Descriptive and summary statistics

All new and previously published ancient DNA sequences were subdivided into the following geographical groups: Iran/Syria, Western Anatolia, Southeastern Europe, Southeastern Central Europe, Italy, Southern France, Central/Western Europe, and Spain. These groups were further subdivided into chronological subgroups reflecting up to four different Neolithic and post-Neolithic periods per region (see Additional file 4 for detailed information on the groupings). Modern sequences were grouped according to their country of origin (also see Additional file 2).

For statistical analyses, all ancient sequences were cut to a 399 bp fragment to match the fragment sizes of previously published ancient sequences (positions 15,914-16,312 according to reference sequence GenBank V00654). Haplotype diversity, mean number of pairwise differences, Tajima’s D, Fu’s Fs and population pairwise F_ST_ were calculated using Arlequin 3.5.1.2 [31]. P values are based on 10,000 random permutations. The level of missing data allowed was adjusted in order to include all nucleotide positions even if there were gaps in some ancient sequences. Besides that, default values were used.

The MDS (multidimensional scaling) plot is based on F_ST_ values calculated using Reynolds’ genetic distances and running 10,000 permutations in Arlequin 3.5.1.2. The MDS plot was created in R 2.14.2 R [32] using the packages MASS [33], plotrix [34] and shape [35].

Correlation between genetic and geographical distances among defined populations/groups was assessed by a Mantel test [36] under 9,999 random permutations using GENALEX 6.4 [37]. The Mantel test is based on F_ST_ values calculated using Reynolds’ genetic distances and running 10,000 permutations in Arlequin 3.5.1.2. Geographical coordinates were determined by eye as the centre of appropriate countries per group for the modern samples and the centre of all archaeological sites per group for the ancient samples.

### Coalescent simulations

Coalescent simulations were performed using Bayes Serial SimCoal [38], by extending the model previously described in Bollongino *et al.* [23]. Similarly, we assume an intergeneration time of 6 years, an ancestral Near Eastern wild aurochs female effective population size of 45,000 [39] and, again, a single domestication process of parameterized size *N*_*D*_ at time 8,500 years BCE (i.e. 1,750 generations BP). Following the domestication bottleneck, this Near Eastern population grows exponentially to a modern Near Eastern and Anatolian effective population size *N*_*NE*_ of 1,007,170 (see SI Bollongino *et al.* [23]). At 6,400 years BCE (i.e. 1,400 generations BP) a proportion of the population *P* is allowed to migrate to form a new and separate European population, which then grows exponentially to a modern European effective population size *N*_*E*_ of 7,942,392 (additional simulations which allow both *N*_*NE*_ and *N*_*E*_ to vary by an order of magnitude are described further in Additional file 5). From the split time until 5,000 years BCE migration between the two populations is allowed at per generation rate *M*_*E*_ (‘early migration’), after which it is changed to rate *M*_*L*_ (‘late migration’). Prior values for *N*_*D*_ are drawn uniformly from the range 1 – 1,000, *P* uniformly from the range 0 – 1, and both migration parameters uniformly from the range 0 – 0.01. The mutation rate is fixed at 45% per million years, the posterior modal value previously estimated by Bollongino *et al.* [23].

We used the above-mentioned 597 previously published modern sequences of 240 bp length (positions 16,023-16,262 according to reference sequence GenBank V00654) and cut the ancient sequences accordingly. The resulting 790 sequences were grouped into 4 sample groups: ancient Near Eastern and Anatolian (*n* = 24), ancient European (*n* = 169), modern Near Eastern and Anatolian (*n* = 100) and modern European (*n* = 497). We calculated 5 within- and 2 between-sample summary statistics (total = 32, also see Additional file 5 for details), and used approximate Bayesian computation (ABC [40]) to estimate parameter values.

## Results

Out of 150 newly analysed bones and teeth from prehistoric domesticated cattle, 113 yielded replicable and highly reliable mitochondrial HVR1 sequences, constituting a success rate of 75.3%. The sequences have been deposited in GenBank [GenBank: KF307209 to KF307322]). None of the blank controls contained amplifiable amounts of bovine DNA (for further detailed discussion of the validity of the ancient DNA data see Additional file 6). The successfully analysed samples come from Bosnia-Herzegovina (3 of 5), Bulgaria (52 of 68), France (15 of 19), Germany (4 of 4), Italy (5 of 14), Romania (15 of 16), Syria (1 of 1), and Turkey (18 of 23).

Using the nomenclature of Achilli *et al.* [41], all sequences belong exclusively to lineages from haplogroups that have previously been defined in present-day European domesticated cattle, namely T3 (n = 70), Q (n = 33), T2 (n = 6) and T, T5 or T1’2’3 (n = 4). None of them belongs to a specific mtDNA motif referred to as haplogroup P that is dominating in the indigenous aurochs population of Europe [26,27,42]. It is of note that the high frequency of haplogroup Q in ancient Southeastern Europe (between 50% and 29% in 5,500-5,000 BCE and 2,700-2,200 BCE, respectively) does not match present-day haplogroup distributions of taurine cattle from Europe, and particularly from the same area (combined frequency for T and Q in present-day Balkan and Greece: 1.5 - 2% [43]). It is also markedly higher than in all other ancient European groups (e.g. only 4% in Central/Western Europe (5,400-4,400 BCE)). See Additional file 4 for detail on haplogroup composition and frequency of haplogroup Q across the 13 spatiotemporal groups.

There are 35 different mitochondrial lineages in the 193 prehistoric individuals, eight of which occur more than once in the dataset. Non-unique haplotypes (H) were named according to their haplogroup and numbered consecutively (H1-H8). Additional files 4 and 7 provide a detailed overview on the distribution of haplogroups and shared and unique haplotypes across the 13 spatiotemporal groups. Only haplotypes called T3_H1, Q_H4, and T2_H7 occur more than twice in the dataset (114, 37, and 5 times, respectively), with T3_H1 also being predominant in present-day taurine cattle. Haplotype T3_H1 occurs in all of the ancient 13 spatiotemporal groups, Q_H4 in all except Spain 2,700-1,600 BCE and Southern France 5,500-4,500 BCE. It is of note that Q_H5, T_H6, and T2_H7 are restricted to the geographical groups of Iran/Syria and Southeastern Europe (Q_H5 in Iran/Syria 4,000-1,400 BCE and Southeastern Europe 6,200-5,500 BCE; T_H6 in Iran 7,000-5,000 BCE and Southeastern Europe 2,700-2,200 BCE; T2_H7 in Iran 7,000-5,000 BCE and Southeastern Europe 5,500-5,000 BCE).

### Genetic distances between cattle populations

**Figure 1:**
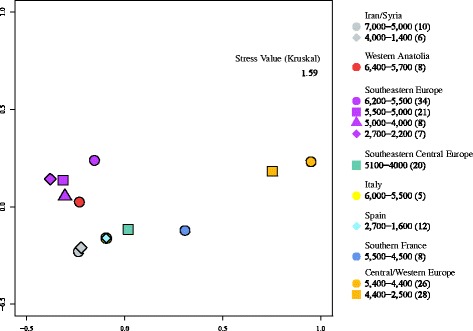
MDS Plot of d-loop sequences from 13 spatiotemporal groups of ancient domesticated cattle. The MDS plot is based on Reynolds’ F_ST_. Numbers represent the age of samples in BCE per group; brackets contain the number of sequences per group.

The MDS plot (Figure 1) reveals a pattern that separates three geographical groups: The four Southeastern European groups cluster with the one from Western Anatolia; both groups from Iran/Syria and from Central/Western Europe are close to each other. However, Southern France and Central/Western Europe are isolated from all other groups and from each other.

**Figure 2:**
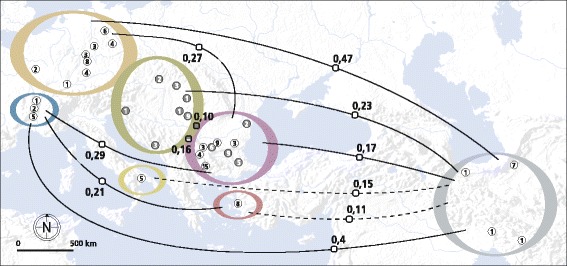
Population pairwise F_ST_s between d-loop sequences from eight Neolithic groups of ancient domesticated cattle. Coloured rings surround geographical groups. Grey and white dots within the circles represent geographical location of archaeological sites. White dots stand for the oldest Neolithic samples per group, grey dots for Middle/Late Neolithic samples. Numbers within dots represent the number of d-loop sequences per site. Orange: Central/Western Europe 5,400-4,400 BCE, blue: Southern France 5,500-4,500 BCE, green: Southeastern Central Europe 5,100-4,000 BCE, yellow: Italy 6,000-5,500 BCE, purple: Southeastern Europe 6,200-5,500 BCE and 5,500-5,000 BCE, red: Western Anatolia 6,400-5,700 BCE, and grey: Iran 7,000-5,000 BCE. Numbers on the lines between coloured circles are population pairwise F_ST_s. Solid lines stand for significant F_ST_s at the 0.05 level, dashed lines stand for significant F_ST_s at the 0.1 level. Grey and white colours of squares on the lines encode which chronological groups per geographi groups are being compared.

Subgroups comprising only the earliest Neolithic cattle of each geographical group were used to further evaluate the influence of sample age and geographical location on genetic distances. Figure 2 maps significant pairwise F_ST_ values between the resulting eight groups. The greatest genetic distances can be observed between Iran 7,000-5,000 BCE and the groups from Central/Western Europe 5,400-4,400 BCE and Southern France 5,500-4,500 BCE with values of 0.47 and 0.40, respectively. These groups also show the greatest geographical distances. The second highest F_ST_ values occur between Southeastern Europe 6,200-5,500 BCE and Central/Western Europe 5,400-4,400 BCE and between Southeastern Europe 6,200-5,500 BCE and Southern France 5,500-4,500 BCE (0.27 and 0.29 respectively). In comparison, the genetic distance between Iran 7,000-5,000 BCE and Southeastern Central Europe 5,100-4,000 BCE is – despite greater geographical distance - slightly lower (0.23). The F_ST_ between Iran 7,000-5,000 BCE and Southeastern Europe 6,200-5,500 BCE is even smaller (0.17). The geographically adjacent groups of Southeastern Europe 6,200-5,500 BCE and 5,500-5,000 BCE and Southeastern Central Europe 5,100-4,000 BCE reveal a distance as high as 0.16 and 0.10, respectively.

A Mantel Test on the basis of Reynolds’ F_ST_ resulted in a strong positive correlation (Rxy: 0.75, P-value 0.001) between geographical and genetic distance among the eight groups. Approximately 56% of the variation can be explained by geographical distance (R^2^ = 0.56). There is a weaker correlation of genetic and geographical distances in modern samples (Rxy: 0.54, P-value 0.002). Here, only 29% of the variation can be explained by geographical distance (R^2^ = 0.29). Complete population pairwise F_ST_ matrices can be found in Additional file 8.

### Measurements of molecular diversity (Ĥ, π), Tajima’s D and Fu’s Fs

The estimates of haplotype diversity (Ĥ), the mean number of pairwise differences (π), Tajima’s D, and Fu’s Fs are given in Table 1.

**Table 1:** **Summary statistics of d-loop sequences from 13 spatiotemporal groups of ancient domesticated cattle**

**Ancient samples**	**# Sequences**	**# Haplotypes**	**Ĥ**	**π**	**Tajima's D ** **(P-value)**	**Fu's Fs ** **(P-value)**
**IR 7,000-5,000 BCE**	10	8	0.96 +/- 0.06	2.82 +/- 1.62	−0.49 (0.33)	**−3.77 (0.01)**
**IR/S 4,000-1,400 BCE**	6	4	0.80 +/- 0.17	3.80 +/- 2.22	−0.79 (0.27)	0.67 (0.59)
**IT 6,000-5,500 BCE**	5	2	0.40 +/- 0.24	0.80 +/- 0.68	−0.97 (0.20)	1.04 (0.63)
**SECE 5,100-4,000 BCE**	20	6	0.52 +/- 0.13	1.30 +/- 0.85	−1.12 (0.13)	−1.48 (0.14)
**SEE 6,200-5,500 BCE**	34	6	0.62 +/- 0.05	1.26 +/- 0.82	0.08 (0.58)	−0.79 (0.32)
**SEE 5,500-5,000 BCE**	21	7	0.78 +/- 0.06	1.79 +/- 1.08	0.12 (0.57)	0.37 (0.60)
**SEE 5,000-4,000 BCE**	8	4	0.79 +/- 0.11	2.11 +/- 1.31	−0.42 (0.37)	0.26 (0.54)
**SEE 2,700-2,200 BCE**	7	5	0.81 +/- 0.13	1.62 +/- 1.08	−0.04 (0.47)	−0.54 (0.22)
**SF 5,500-4,500 BCE**	8	1	0.00 +/- 0.00	0.00 +/- 0.00	-	-
**SP 2,700-1,600 BCE**	12	4	0.45 +/- 0.17	1.30 +/- 0.87	**−1.71 (0.04)**	−0.05 (0.47)
**WA 6,400-5,700 BCE**	8	4	0.64 +/- 0.18	0.79 +/- 0.63	**−2.14 (0.00)**	**−1.73 (0.02)**
**CWE 5,400-4,400 BCE**	26	4	0.22 +/- 0.11	0.45 +/- 0.41	**−1.53 (0.03)**	−0.24 (0.32)
**CWE 4,400-2,500 BCE**	28	9	0.50 +/- 0.12	0.91 +/- 0.65	**−2.08 (0.01)**	**−4.55 (0.00)**

IR/S: Iran/Syria, IT: Italy, SECE: Southeastern Central Europe, SEE: Southeastern Europe, SF: Southern France, SP: Spain, WA: Western Anatolia, and CWE: Central/Western Europe. Numbers in the first column indicate the age of the samples in BCE. Ĥ: Haplotype diversity, π: mean number of pairwise differences. Significant Tajima’s D and Fu’s Fs value at the 0.05 level are highlighted in bold.

Haplotype diversity clearly decreases in a southeast to northwest direction with Iran 7,000-5,000 BCE (0.96) at the high end, and Southern France 5,500-4,500 BCE (0.00) and Central/Western Europe 5,400-4,400 BCE (0.22) at the low end. The haplotype diversity of the earliest domesticated cattle on the European continent in Southeastern Europe 6,200-5,500 BCE (0.62) is much lower than in Iran, and comparable to Western Anatolia 6,400-5,700 BCE (0.64), but higher than in the geographically close European group of Southeastern Central Europe 5,100-4,000 BCE (0.52). Again, diversity in Central/Western Europe 5,400-4,400 BCE is substantially lower (0.22). Following the northern Mediterranean coast, the values also drop sequentially from Western Anatolia 6,400-5,700 BCE (0.64) to Italy 6,000-5,500 BCE (0.40) to Southern France 5,500-4,500 BCE (0.00).

Haplotype diversity estimates increase with time in the two regions where samples from two Neolithic periods are available: from 0.22 to 0.50 in Central/Western Europe and from 0.62 to 0.78 in Southeastern Europe. In Southeastern Europe, haplotype diversity remains the same in the subsequent Chalcolithic group 5,000-4,000 BCE (0.78), but increases again during the Bronze Age 2,700-2,200 BCE (0.81). Similar patterns are observed when considering the mean numbers of pairwise differences. The Neolithic subgroups also show a tendency of decreasing values with distance from Iran. Regionally, the values increase with time; in Central/Western Europe from 0.45 to 0.91 and in Southeastern Europe from 1.26 to 1.79 to 2.11. In the youngest Southeastern European group (2,700-2,200 BCE) the value drops again (0.54). In Central/Western Europe, where both diversity indices increase with time, Tajima’s D is also significantly negative (Fu’s Fs only in the younger group). This is not the case for Southeastern Europe. Diversity estimates of the 597 modern cattle sequences only show a slight tendency towards an east to west gradient for both haplotype diversity, and the mean number of pairwise differences. Tajima’s D and Fu’s Fs are mostly significantly negative. All diversity estimates and graphical visualisations of chronological and geographical diversity trends can be found in the Additional file 9.

### Coalescent simulations

**Figure 3:**
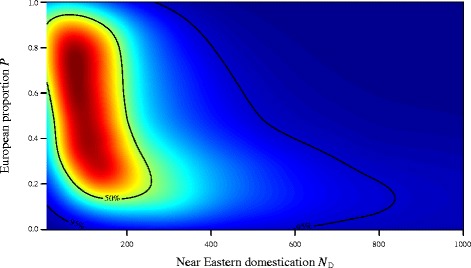
Joint posterior density for the domestication bottleneck (*N*_*D*_) and the proportion moving into Europe (*P*). The approximate joint posterior probability density of the proportion of the population *P* allowed to move into Europe at the time of the split (6,400 BCE) and the effective female population size at the time of the domestication event (*N*_*D*_). The 50% and 95% credible intervals are overlaid as contours.

**Figure 4:**
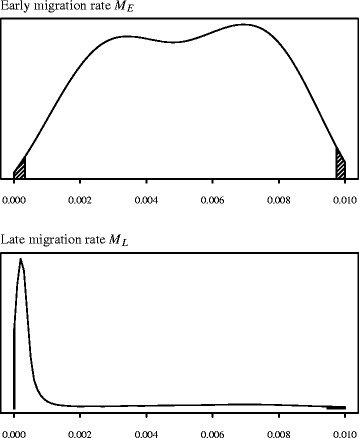
The marginal approximate posterior probability densities of the two migration rate parameters. *M*_*E*_ (‘early migration’) is the rate from the population split time until 5,000 BCE, and *M*_*L*_ (‘late migration’) is the rate from 5,000 BCE to present.

We performed 5 million coalescent simulations under the demographic model described above, and used a tolerance proportion of 0.1%, meaning that we retained the 5,000 best parameter sets. Figure 3 shows the joint posterior density of parameters *N*_*D*_ and *P* (marginal to the remaining two), with the joint mode found at *N*_*D*_ = 81 and *P* = 0.73. The marginal modal value for *N*_*D*_ was 92 (95% credible interval: 29 – 783). Marginal densities for the two migration parameters *M*_*E*_ and *M*_*L*_ are given in Figure 4. While it is not possible to infer much from the relatively uninformative posterior for *M*_*E*_ (top, mode 0.006 94; 95% CI: 0.00033 – 0.00974), we are able to say that migration between the Near East and Europe (*M*_*L*_) appears to have been greatly reduced, essentially to zero, in the period after 5,000 years BCE (bottom, mode 0.00022; 95% CI: 0.00001 – 0.00946). Further simulations were performed in order to test the sensitivity of these parameter estimations to our assumed fixed values of *N*_*NE*_ and *N*_*E*_. Increasing or decreasing both *N*_*NE*_ and *N*_*E*_ by an order of magnitude produced estimates that did not significantly differ from those given above (see Additional file 5 for details).

## Discussion

### The domestication process of taurine cattle

Using an ancient (*n* = 193) and modern mtDNA dataset (*n* = 597) of domesticated cattle from the Near East, Anatolia and Europe for coalescent simulation and approximate Bayesian computation, we inferred a (joint) posterior mode of 81 female founder individuals at the beginning of the domestication process. This result is consistent with the previous estimate based on 15 ancient Iranian and 27 modern Near Eastern and Anatolian cattle [23], and demonstrates that this initial finding of a very strong Near Eastern bottleneck is robust even with a greatly expanded continent-wide data set, and is not biased by theoretically possible subsequent introgression from aurochs populations outside the Near East. It can therefore be concluded that domestic cattle indeed have a discrete and rather localised origin, very likely in Southeastern Anatolia and the Near East, a view that is consistent with a huge body of archaeozoological evidence from the 9^th^ millennium BCE [44-46].

Subsequent to the first domestication phase, the ancient DNA data, together with archaeological evidence, point to an intermittent expansion scenario. Expanding from Southeastern Anatolia, cattle reached Western Anatolia and the Aegean not before 7,000 BCE. From here, they spread simultaneously across Southeastern Europe and along the Mediterranean coast into Central, Northern, Southwestern, and Western Europe. In essence, the observed strong correlation between genetic and geographical distances together with decreasing genetic diversity roughly in a southeast to northwest and southwest direction is consistent with the idea of serial dilution of diversity by a series of recurring founder events. The oldest (Neolithic) groups with the greatest geographical distance from each other, namely Iran and Central/Western Europe and Southern France, show the highest F_ST_ values (0.47 and 0.4, respectively). Smaller genetic distances are observed between more adjacent areas, such as between Iran and Western Anatolia and between Iran and Southeastern Europe (0.11 and 0.17, respectively). Other statistics are equally consistent with the serial dilution model: Neolithic cattle from Iran yield the highest value for haplotype diversity in the whole dataset (0.96). Haplotype diversity consistently decreases along the proposed two main Neolithisation routes, with the lowest values in remote areas, i.e. in Neolithic Central/Western Europe and Southern France (0.22 and 0.00, respectively), while intermediate values are observed in between.

Alternative scenarios of secondary domestications or traceable female gene-flow from wild aurochs in Europe have been discussed several times in the literature [29,47-51]. The arguments are mainly based on scarce findings of the mtDNA haplogroup P, pre-dominating in European aurochs, in the domesticated stock [49,50] on the one hand, and the presence of mtDNA lineages in pre-Neolithic Italian aurochs that resemble those of the imported domesticated animals [29,48] on the other, thereby impeding the detection of introgression by mere comparison of haplogroup composition. However, realistic expectations under such models would also include i) a larger inferred founder population due to introgressions of diverse aurochs lineages and ii) significant deviations from the serial dilution of genetic diversity model. None of the two has been observed in or can be inferred from the data presented here. Detection of potential introgression of Italian aurochs through time deserves further attention, e.g. by expanding the existing dataset to encompass finds from diverse archaeological sites and later chronological phases. However, the existing dataset from the rest of Europe suggests that introgressions of local genes into the imported domestic cattle populations are rare and geographically restricted exceptions, or coming from male aurochs. Separate independent domestication(s) of European aurochs can almost with certainty be excluded.

The strict separation of domestic cattle from their wild European relatives is very different to what can be observed in other animals. For example, pigs were imported to Europe in a similar way to cattle, but after a few centuries all their mitochondrial lineages were replaced through admixture with local wild boar [52,53].

### The first domesticated cattle in Europe

The summary statistical patterns described here may be partly biased by the fact that the analysed data come from heterochronous and spatially diverse samples [54]. Therefore, we used coalescent simulations to estimate the key parameters of taurine cattle population history upon their arrival in Europe in a realistic evolutionary demographic framework.

Our model suggests that a high proportion (73%) of domesticated cattle in Anatolia and the Near East may have migrated into Europe. This indicates that the expansion into Europe was a far less severe bottleneck than assumed, and that much of the variation present in the original Anatolian/Near Eastern population survived in initial European cattle populations. Consistent with this, the Western Anatolian and Southeastern European sample groups constitute a cluster in the MDS plot (Figure 1). However, Southern France and Central/Western Europe instead are clearly separated, very likely reflecting genetic diversification along the two main Neolithisation routes. It is noteworthy that the data from Western Anatolia, Southern Italy, and Southern France come from very few sites with less than ten samples each (eight, five, and eight, respectively) and therefore have to be evaluated cautiously. However, the drastic decline in haplotype diversity and mean number of pairwise differences from 0.64/0.80 and 0.40/0.79 in Western Anatolia and Italy to 0.00/0.00 in Southern France is a good fit to a scenario of only few individuals being transported by boat to the Northwestern Mediterranean coast [3,55]. Low diversity estimates are also congruent with the fact that cattle did not play a major role in the domesticated faunal spectrum of Neolithic economies from Mediterranean Europe (Impressa and Cardial), in contrast to Neolithic Cultures in Central Europe, where domesticated cattle were generally well represented [56,57].

Tracing the spread of cattle through the European mainland, there are patterns in the data that point to significant demographic changes connected to the expansion of the Neolithic culture from Southeastern to Central/Western Europe. The genetic distance between Southeastern Europe (6,200-5,500 BCE) and Central/Western Europe (5,400-4,400 BCE) is unexpectedly high (0.27). To put this high value into context: the F_ST_ values between Iran (7,000-5,000 BCE), Southeastern Europe (6,200-5,500 BCE) and Italy (6,000-5,500 BCE) are much lower (0.17 and 0.15, respectively) despite larger geographic distances. A good indicator for this massive demographic change is that the frequency of the mitochondrial Q-lineage drops from 50 % in Southeastern European (6,200-5,500 BCE) to 4% in Central/Western Europe (5,400-4,400 BCE). Haplotype diversity decreases drastically from 0.62 to 0.22. There are several additional lines of evidence that point to the region between Southeastern Europe and Central Europe as a kind of core area where the Neolithic idea was re-consolidated: i) From archaeology: The Linearbandkeramik culture (LBK, engl. Linear Pottery culture) developed here and spread rapidly over Central Europe starting around 5,600 BCE [58]; ii) From palaeogenetics: A migration of farmers from Southeastern to Central Europe has been inferred using ancient mtDNA [18]; iii) From gene-culture coevolutionary modelling: Spatially-explicit computer simulations of the spread of an allele associated with lactase persistence in humans (i.e. the ability to digest milk sugar as an adult), point to this area as where positive selection started affecting the frequency of this allele in dairying cultures [59]. We therefore suggest that the observed substantial loss of genetic diversity and the increasing genetic distance in prehistoric cattle are the result of a significant founder event along with the spread of the LBK. It probably coincides with a major wave of human migration and is followed by a period of intensified cattle breeding resulting in a rising importance of dairying. This picture becomes even more comprehensive when we look at how patterns change after the early Neolithic.

### After the arrival

Cattle herding becomes more and more important with the onset of the LBK. A few centuries later, cattle bones constitute up to 70% of all domesticated animal bones in faunal assemblages in Central Europe, a value that stays roughly the same for most of the subsequent millennia with some regional fluctuations [56,57]. Accordingly, significantly negative Tajima’s D and Fu’s Fs values in Neolithic Central/Western Europe and in the majority of the modern sample groups point to extended periods of population growth (see Table 1 and Additional file 9).

Interestingly, there is no indication for population growth in Southeastern Europe. The observed diachronic increase in haplotype diversity in the Southeastern European sample groups appears in tandem with new, previously absent mitochondrial haplotypes (also see Additional file 7). It is of note that two of these new haplotypes (T_H6 and T2_H7) are present here and in the Iranian Neolithic sample but not elsewhere.

According to our demographic modelling, migration between Anatolia/the Near East and Europe was greatly reduced, essentially to zero, in the period after 5,000 BCE. We should expect that accurately estimating the level of early migration between 6,400 and 5,000 BCE to be difficult, as it is somewhat confounded by the proportion of cattle *P* moving to Europe at the time of the split (indeed the two parameters are very slightly negatively correlated; degree *r* = -0.07, *p* = 0.0002). However, it is clear that there is support at least for *some* level of migration during this early period as the estimated modal migration rate is clearly greater than 0.

We therefore suggest that a probable underlying scenario for our observations is one of continuous gene-flow into Europe following the initial colonization at around 6,400 BCE. This scenario also fits in with archaeological evidence for accelerated westward acculturation occurring in the first half of the 6^th^ millennium BCE [6,60]. This early phase was followed by almost total isolation between the European and Anatolian/Near Eastern cattle populations after 5,000 BCE.

It is of note that this pattern has changed again in later periods. The pattern of decreasing diversity in the direction of the Neolithic expansion and the correlation of genetic and geographical distances is considerably weaker in modern-day cattle breeds than in the Neolithic. It is not clear yet to which extent human migrations from the East as postulated for the Bronze Age [61] influenced the already existing cattle stock in Europe. However, the fading geographical patterns are likely mirroring more recent demographic changes and founder events, such as global trade, exceptional selection pressure on particular high performance breeds and replacement of traditional breeds [62]. Thus, the present study explicitly underlines that ancient demographic and evolutionary processes in selectively bred animals can only be uncovered by using ancient DNA data.

## Conclusions

Overall, palaeogenetic together with archaeological and archaeozoological data strongly support the following scenario: taurine cattle were domesticated in a region between Southeastern Anatolia and the Zagros Mountains, Syria and the Lebanon. The domestication process started in the mid-9^th^ millennium BCE, with a small effective number of wild female aurochs (estimated modal value of 81). After 7,000 BCE, domestic cattle populations were transported from the Central Anatolian plateau to Western Anatolia and the Aegean. Much of the original Anatolian and Near Eastern variation (approximately 73%) survived in the first Neolithic cattle that were introduced to Europe around 6,400 BCE. Despite some evidence for subsequent gene-flow with Anatolia and the Near East between 6,400 and 5,000 BCE, most of the initial genetic diversity was lost as cattle spread through Europe along with the Neolithic transition: Via the Mediterranean trajectory, migrating farmers reached i.e. Southern Italy, Northern Africa, the Tyrrhenian Islands, Southern France and the Iberian Peninsula by boat. The low genetic diversity observed in the few genetic data available from these regions points to a significantly low effective population size of cattle arriving in the Western Mediterranean. Along the second trajectory across the European mainland and without major signs of introgression from wild aurochs, cattle finally reached Central, Western (after 5,500 BCE) and Northern Europe (after 4,100 BCE). Also here, much of the genetic diversity was lost during the move, particularly when cattle were brought to Central Europe by LBK farmers.

Gene-flow between Europe and Anatolia and the Near East appears to have been reduced, essentially to 0, after around 5,000 BCE. In modern breeds however, the genetic effects of the inferred migratory patterns and geographical diversification become far less pronounced, probably due to selective breeding and trade of high performance cows in very recent times.

In summary, the genetic prehistory of domestic cattle seems to consist of a small, localised domestication process, followed by a relatively straightforward series of spasmodic expansion episodes resulting in a serial dilution of genetic diversity from the Near East to Western and Northern Europe. Future genomic multi-locus studies of ancient DNA from prehistoric periods will hopefully add greater detail to this picture, particularly by incorporating the potentially divergent demography of male cattle.

## **Additional files**

Additional file 1:
**Sample list.** Supplemental information on the location of the archaeological sites, dating, sample providers, sequencing results, and GenBank accession numbers of all new sequences. Additional file 2:
**GenBank accession numbers.** GenBank accession numbers and references of all previously published ancient and modern mtDNA sequences used in this study. Modern sequences are grouped geographically by their country of origin. Additional file 3:
**Ancient DNA methods.** Supplemental methodological detail on DNA amplification and DNA sequencing (such as primer sequences and locations, and detailed protocols), haplogroup determination, and establishment of consensus sequences. Additional file 4:
**Chronological and geographical sample groups.** Individual assignment of all ancient sequences used in this study to chronological, cultural and geographical groups, and individual haplogroup and haplotype assignments and polymorphic positions. The relative frequency of halplogroup Q per group is provided with a 95% confidence interval (CI). Shared haplotypes (H) are numbered consecutively. Haplogroup T stands for T, T5 or T1'2'3. Haplogroup assignment according to [41]. Polymorphic positions are given according to the reference sequence GenBank V00654, whereby dots represent bases that match the reference sequence, and asterisks unavailable sequence information. Additional file 5:
**Coalescent simulations and ABC.** Methodological detail on the coalescent-based demographic modelling of the domestication and then spread of cattle into Europe. Additional file 6:
**Validation of ancient DNA data.** Supplemental information on ancient DNA validity criteria (such as blank controls and contamination rate estimation), and explicit discussion of not fully replicated sequences. Additional file 7:
**Shared haplotypes.** Shared haplotypes (H) are named and coloured according to their haplogroup and numbered consecutively. Shades of red: T3; White/light gray: Q; Shades of blue: T, T5 or T1’2’3; Shades of green: T2 (haplogroup definition according to [41]). The x-axis gives the number of sequences. a) Haplotype distribution across all 193 ancient mtDNA sequences; b) Haplotype distribution across 13 spatiotemporal groups defined by region of origin and age in BCE to the left of each bar. Additional file 8:
**F**_**ST**_ **values.** a) Ancient pairwise F_ST_s. b) Ancient pairwise F_ST_ P values. c) Ancient Reynolds’ F_ST_s. d) Modern pairwise F_ST_s. e) Modern pairwise F_ST_ P values. For a)-c): First row/column:IR/S: Iran/Syria; IT: Italy; SECE: Southeastern Central Europe; SEE: Southeastern Europe; SF: Southern France; SP: Spain; WA: Western Anatolia; CWE: Central/Western Europe.Numbers indicate the age of the samples in BCE; numbers in brackets indicate sample size.Significant values at the 0.05 level are highlighted in bold. For d)-e): First row/column: Area/country of origin. Numbers in brackets indicate sample size. Significant values at the 0.05 level are highlighted in bold. Additional file 9:
**Graphical representation of Table 1 and summary statistics from modern mtDNA sequences.** a) Boxplots of ancient haplotype diversity. b) Boxplot of ancient mean numbers of pairwise differences. Estimates are sorted by the magnitude of the oldest available chronological group per geographical group. Markers are colored by chronology as follows: white: (earliest) Neolithic, grey: Middle/Late Neolithic, dashed: Chalcolithic, black: post-Neolithic. X -axis: IR/S: Iran/Syria, IT: Italy, SECE: Southeastern Central Europe, SEE: Southeastern Europe, SF: Southern France, WA: Western Anatolia, and CWE: Central/Western Europe. Numbers indicate the age of the samples in BCE; numbers in brackets indicate sample size. c) Summary statistics of d-loop sequences from 11 geographical groups of modern domesticated cattle. First column: area/country of origin. Ĥ: Haplotype diversity, π: mean number of pairwise differences. Significant Tajima’s D and Fu’s Fs value at the 0.05 level are highlighted in bold. d) Boxplot of modern haplotype diversity. e) Boxplot of modern mean numbers of pairwise differences. The boxplots are sorted by magnitude. X-axis: country/area of origin; numbers in brackets indicate sample size.
