# Supplementary material for: The genetic prehistory of domesticated cattle from their origin to the spread across Europe
Source: BMC Genet. 2015 May 28;16:54. doi: 10.1186/s12863-015-0203-2 (PMC4445560; doi:10.1186/s12863-015-0203-2)
Supplement: Additional file 3: — Ancient DNA methods. Supplemental methodological detail on DNA amplification and DNA sequencing (such as primer sequences and locations, and detailed protocols), haplogroup determination, and establishment of consensus sequences. [file 12863_2015_203_MOESM3_ESM.pdf]

# Additional file 3

**1. DNA Amplification** **p.1**

**2. DNA sequencing** **p.6**

**3. Establishment of consensus sequences** **p.6**

## 1. DNA amplification

Amplification of the HVSI from position 15914 - 9 (reference sequence GenBank V00654) was conducted using PCR primers that produce six overlapping fragments (hereinafter referred to as standard primer set). For 23 samples where amplification failed using the standard set, two alternate primer pairs were created that reduce amplicon length in favour of better amplification success. Primer sequences, positions, and annealing temperatures are given in Table 1. Figure 1 provides visualisation of the HVSI amplification strategy.

In 30 of 33 cases when a particular d-loop motif characteristic of this rare mtDNA clade was found (15,953 G and 16,255 C [1]), two additional amplicons outside the HVSI each covering a transversion (3,415 A and 10,717 T [1]) were amplified and sequenced. These additional fragments were also retrieved from eight previously published samples showing this motif and using remaining DNA extract (Ap7, God2, Mar10, Mf7, Sac3, Sac5, Sac7, Zag1 [2-4]). All of them, but none of the 14 other randomly chosen sequences from different haplogroups, show a full association with these transversions (see Table 2). We infer from this result that 15,953 G and 16,255 C alone can be considered as characteristic for haplogroup Q.

New primers were developed using the program PrimerSelect™ (DNASTAR Lasergene® 7.1 and 8).

***Table 1: Mitochondrial primers.***

*3’ end positions are given according to reference sequence V00654. The names indicate upper (U) and lower (L) primer. The standard primer set covering the targeted HVSI region in six overlapping fragments is in bold. Primers U1b and L2c are alternatives to U1 and L2 with shorter amplicon length. U8 is an alternative to U4 and replaced the latter in the course of this study because of its better performance. Primer pairs A8U/A7L and A9U/A8L are alternatives to U5/L5 with shorter amplicon length. Additionally, both primers were designed specifically not to amplify Sus scrofa DNA due to mismatches at the 3’ ends. As Sus scrofa DNA is a possible lab contaminant because of its presence in various reagents [5, 6], these alternative primers were used in the rare cases when the rather unspecific primer pair U5/L5 had first resulted in Sus scrofa sequences.*

| Name | Sequence in 5’-3’-direction | 3‘ end position | Primer length | Amplicon length | Annealing temperature | Reference |
|---|---|---|---|---|---|---|
| **BosU1** | **CCAAATATTACAAACACCACTAGCTAAC** | 15913 | 28 bp | | | |
| **BosL1** | **GCATGGGGCATATAATTTAATGTACT** | 16005 | 26 bp | 91 bp | | [4] |
| BosU1b | GCTAACATAACACGCCCATACAC | 15930 | 23 bp | 74 bp | 56°C | this study |
| **BosU2** | **AATGTACATAACATTAATGTAATAAAGACA** | 15992 | 20 bp | | | |
| **BosL2** | **TTGACATAATGTACTATGTACAGTCAATAA** | 16077 | 30 bp | 84 bp | 52°C | [4] |
| BosL2c | TCATGTACTTGCTTATATGCATGG | 16025 | 24 bp | 32 bp | 56°C | this study |
| **BosU3** | **CCATGCATATAAGCAAGTACATGA** | 16048 | 24 bp | | | |
| **BosL3** | **CTCGTGATCTAATGGTAAGGAATATATA** | 16131 | 28 bp | 82 bp | 52°C | [4] |
| **BosU8** | **GTACATTATGTCAAATTCATTCTTGATAG** | 16121 | 29 bp | 80 bp | | this study |
| BosU4 | ACTGTACATAGTACATTATGTCAAATTCAT | 16112 | 30 bp | | | |
| **BosL4** | **CGGAGCGAGAAGAGGGATC** | 16202 | 19 bp | 89 bp | 56°C | [4] |
| BosA8U | GTGAAACCAGCAACCCGCTA | 16195 | 20 bp | | | |
| BosA7L | AATGAATTTTACCAGGCATCTGGTT | 16249 | 25 bp | 53 bp | 56°C | |
| BosA9U | CCATAAACCGTGGGGGTCG | 16242 | 19 bp | | | |
| BosA8L | TCTTCAGGGCCATCTCATCTAAAAC | 16275 | 25 bp | 34 bp | 57°C | this study |
| **BosU5** | **TACCATGCCGCGTGAAACCA** | 16184 | 20 bp | | | |
| **BosL5** | **TGAGATGGCCCTGAAGAAAGAA** | 16272 | 22 bp | 87 bp | 56°C | |
| **BosU6** | **GGTCGCTATCCAATGAATTTTAC** | 16260 | 23 bp | | | |
| **BosL6** | **ATGTGTGAGCATGGGCTGATTA** | 10 | 22 bp | 87 bp | 56°C | [4] |
| BosQ1U | TGAATTCCCCTACCAATACCCTATCCTCTT | 3385 | 30 bp | | | |
| BosQ1L | CATATCAAGCCTAGCCGTATACTCCATTCT | 3421 | 30 bp | 35 bp | 60°C | |
| BosQ4U | CAGTTTGGCGACAACAGCCTTAAT | 10681 | 24 bp | | | |
| BosQ4L | TTTTAACCATATGGCTCCTCCCTCT | 10728 | 25 bp | 46 bp | 58°C | this study |

| Lab Code | 3.415 | 10.717 | Haplogroup | Reference |
|---|---|---|---|---|
| *V00654* | ***A*** | ***T*** | *T3* | |
| Ap7 | **T** | **A** | Q | Bollongino et al. 2006 |
| God2 | **T** | **A** | Q | Bollongino et al. 2006 |
| Mar10 | **T** | **A** | Q | Edwards et al. 2007 |
| Mf7 | **T** | **A** | Q | Bollongino et al. 2012 |
| Sac3 | **T** | **A** | Q | Bollongino et al. 2012 |
| Sac5 | **T** | **A** | Q | Bollongino et al. 2012 |
| Sac7 | **T** | **A** | Q | Bollongino et al. 2012 |
| Zag1 | **T** | **A** | Q | Bollongino et al. 2012 |
| Ap15 | **T** | **A** | Q | this study |
| Ap16 | **T** | **A** | Q | this study |
| Ap17 | | | T3 | this study |
| Ber2 | | | T3 | this study |
| Bor3 | **T** | **A** | Q | this study |
| Cav10 | **T** | **A** | Q | this study |
| Cav11 | **T** | **A** | Q | this study |
| Cav12 | **T** | **A** | Q | this study |
| Dra2 | **T** | **A** | Q | this study |
| Dra3 | **T** | **A** | Q | this study |
| Dra4 | | | T3 | this study |
| Dra7 | **T** | **A** | Q | this study |
| Dra8 | **T** | **A** | Q | this study |
| Dra27 | **T** | **A** | Q | this study |
| Dra30 | | | T2 | this study |
| Kan1 | | | T3 | this study |
| Kan2 | | | T | this study |
| Kan3 | | | T | this study |
| Kan5 | **T** | **A** | Q | this study |
| Kan7 | **T** | **A** | Q | this study |
| Kop1 | | | T3 | this study |
| Kop12 | **T** | **A** | Q | this study |
| Kov22 | **T** | **A** | Q | this study |
| Kov24 | **T** | **A** | Q | this study |
| Kov25 | **T** | **A** | Q | this study |
| Kov26 | **T** | **A** | Q | this study |
| Kov42 | | | T3 | this study |
| Kov43 | **T** | **A** | Q | this study |
| Kov47 | **T** | **A** | Q | this study |
| Kov50 | **T** | **A** | Q | this study |
| Kov53 | **T** | **A** | Q | this study |
| Kov54 | **T** | **A** | Q | this study |
| Oko7 | **T** | **A** | Q | this study |
| Oko8 | | | T3 | this study |
| Ovc6 | **T** | **A** | Q | this study |
| Ovc8 | **T** | **A** | Q | this study |
| Ovc9 | **T** | **A** | Q | this study |
| Pe18 | **T** | **A** | Q | this study |
| Sam2 | **T** | **A** | Q | this study |
| Sam11 | **T** | **A** | Q | this study |
| Sam13 | | | T2 | this study |
| Uiv5 | | | T2 | this study |
| Uiv19 | | | T | this study |
| Uiv20 | | | T | this study |

**Table2: Q specific transversions.**

*Results for two additional positions outside the HVSI characteristic for haplogroup Q (positions 3,415 and 10,717 according to reference sequence GenBank V00654.*

***Figure 1: Mitochondrial primer scheme***

*Dark green and red bars indicate the position of the standard primer set according to the reference sequence (blue bar, GenBank V00645). Bright green and red bars indicate the position of the alternate primer pairs as specified in Table 1. Numbers within bars indicate the name of the primer and the 3' end position. Numbers on the connections indicate sequence length of the amplified region with/without primer sequence.*

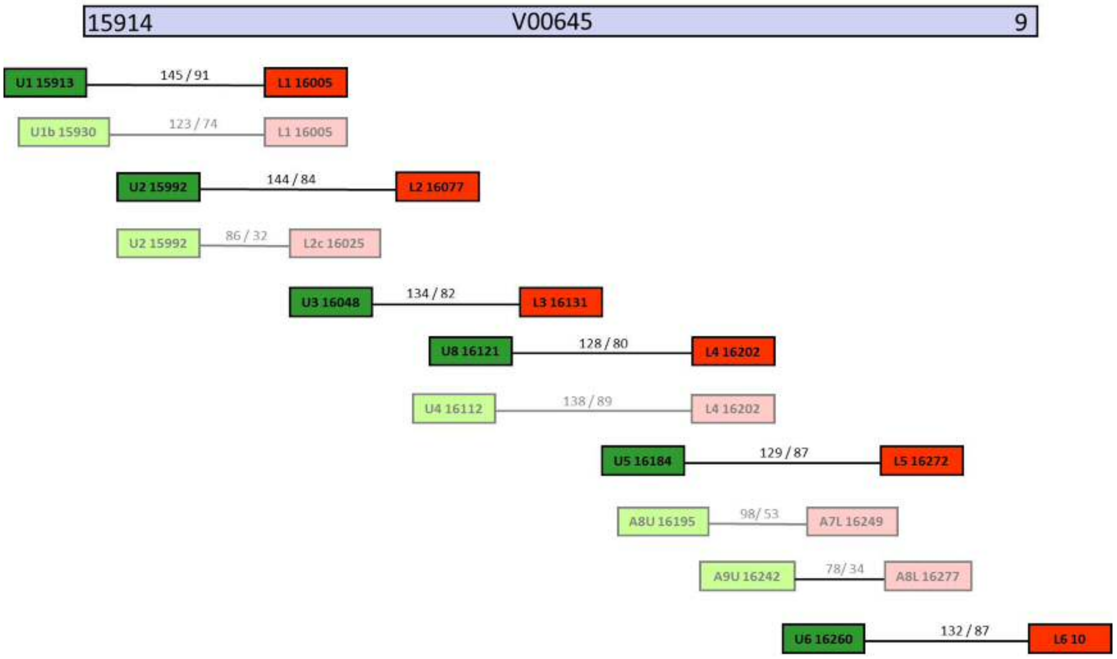


Amplification reactions were set up as shown in Table 3. At least three successful independent PCRs from two extractions were attempted.

Blank controls were processed during each PCR. The enzyme Uracil-N-Glycosilase was added to about 5% of the PCRs to cleave deaminated cytosine residues from particularly damaged samples. Amplification was carried out in a Mastercycler gradient (Eppendorf). PCR products were visualized on an agarose gel by addition of ethidium bromide. PCR products were purified by incubation with the two enzymes Exonuclease I (2 U; Fermentas) and Shrimp Alkaline Phosphatase (0.3 U; Fermentas) for 45 min at 37°C, followed by heat inactivation for 15 min at 80 °C.

***Table 3: PCR reagents and conditions.***

*Standard volumes and cycles are in bold. The protocol was sometimes modified in the given ranges to optimize amplification success. UNG was used only sporadically (see above).*

| **Reagent** | **Volume (µl)** | **Concentration (stock solution)** | **Final concentration** | **PCR parameters** | |
|---|---|---|---|---|---|
| **PCR Gold Buffer (Applied Biosystems)** | 5-**6** | 10 x | 1 x – 1.2 x | **Initial activation** | 6 min 90°C |
| **$MgCl_2$ (Applied Biosystems)** | 4-**6** | 25 mM | 2 mM – 3 mM | | |
| **dNTP's (Qiagen)** | 1 | 10 mM | 0.2 mM | **Denaturation** | 40 sec 94°C |
| **BSA (Roche)** | **1**-2 | 20 mg/ml | 0.4 µg/µl – 0.8 µg/µl | **Annealing** | 40 sec (temp. see primers) |
| **Primer, each (Biospring)** | 1 | 10 µM | 0.2 µM | **Elongation** | 40 sec 72°C |
| **AmpliTaq Gold® (Applied Biosystems)** | **0.5**-1 | 5 U/µl | 2.5 U – 5 U | | |
| **UNG (Invitrogen)** | 0.5 | 1 U/µl | 0.5 U | | |
| **DNA** | 0.5-14 | not determined | not determined | | |
| **UV-irradiated HPLC-$H_2O$ (Acros Organics)** | add 50 | | | **Cycles** | **50**-60 |

## 2. DNA sequencing

Cycle sequencing reactions were set up as shown in Table 4. Products were sequenced by capillary electrophoresis on an ABI PRISM™ 3130 Genetic Analyzer (Applied Biosystems) using POP-6™ polymer (Applied Biosystems).

***Table 4: Cycle sequencing reagents and conditions.***
*Sequencing products were obtained using the BigDye® Terminator v.1.1 chemistry (Applied Biosystems). Volumes of PCR products were adjusted according to the intensity of their bands in the agarose gel.*

| **Reagent** | **Volume (µl)** | **Concentration (stock solution)** | **Final concentration** | **PCR parameters** | |
|---|---|---|---|---|---|
| **Buffer** | 1.5 | **5 x** | **0.75 x** | **Denaturation** | 30 sec 92 °C |
| **BigDye** | 1 | **na** | **na** | **Annealing** | 15 sec (temp. see primers) |
| **Primers (Biospring)** | 1 | **10 µM** | **1µM** | **Elongation** | 2 min 30 sec 60°C |
| **PCR product** | 1-4 | not determined | not determined | | |
| **HPLC-$H_2O$ (Acros Organics)** | add 10 | | | **Cycles** | 25 |

## 3. Establishment of consensus sequences

Sequences were further analysed using the programs SeqMan™ and MegAlign™ (DNASTAR Lasergene® 7.1 and 8). At least three sequences obtained from independent PCRs from two independent DNA extractions per sample per primer pair were usually used to create a majority rule consensus sequence. If divergent positions occurred among the three sequences, we attempted to sequence further PCR products, at best obtained using UNG (see above), until the variable position was present in at least a 3:1 ratio.

**Literature**

1. Achilli A, Olivieri A, Pellecchia M, Uboldi C, Colli L, Al-Zahery N, Accetturo M, Pala M, Hooshiar Kashani B, Perego UA *et al*: **Mitochondrial genomes of extinct aurochs survive in domestic cattle**. *Current Biology* 2008, **18**(4):R157-158.
2. Bollongino R, Edwards CJ, Alt KW, Burger J, Bradley DG: **Early history of European domestic cattle as revealed by ancient DNA**. *Biology Letters* 2006, **2**(1):155-159.
3. Edwards CJ, Bollongino R, Scheu A, Chamberlain A, Tresset A, Vigne J-D, Baird JF, Larson G, Ho SYW, Heupink TH *et al*: **Mitochondrial DNA analysis shows a Near Eastern Neolithic origin for domestic cattle and no indication of domestication of European aurochs**. *Proceedings of the Royal Society B: Biological Sciences* 2007, **274**(1616):1377-1385.
4. Bollongino R, Burger J, Powell A, Mashkour M, Vigne JD, Thomas MG: **Modern Taurine Cattle descended from small number of Near-Eastern founders**. *Molecular Biology and Evolution* 2012:doi:10.1093/molbev/mss1092.
5. Leonard JA, Hofreiter M, Kreuz E, Hodges L, Ream W, Wayne RK, R.C. F: **Animal DNA in PCR reagents plagues ancient DNA research.** *Journal of Archaeological Science* 2007, **34**:1361-1366.
6. Geörg C: **Paläopopulationsgenetik von Schwein und Schaf in Südosteuropa und Transkaukasien. ForschungsCluster1.**, vol. 9. Rahden/Westf: Verlag Marie Leidorf GmbH; 2013.
