# Supplementary material for: The genetic prehistory of domesticated cattle from their origin to the spread across Europe
Source: BMC Genet. 2015 May 28;16:54. doi: 10.1186/s12863-015-0203-2 (PMC4445560; doi:10.1186/s12863-015-0203-2)
Supplement: Additional file 5: — Coalescent simulations and ABC. Methodological detail on the coalescent-based demographic modelling of the domestication and then spread of cattle into Europe. [file 12863_2015_203_MOESM5_ESM.pdf]

Additional file 5

Coalescent simulations and ABC

The 790 DNA sequences were grouped into 4 sample groups: ancient Near Eastern and Anatolian (n = 24), ancient European (n = 169), modern Near Eastern and Anatolian (n = 100) and modern European (n = 497). Within sample (number of haplotypes, number of segregating sites, average pairwise difference, haplotype diversity and Tajima's D) and between sample summary statistics (F_{ST}, average pairwise difference) were calculated on the observed data using Arlequin 3.5.1.2 [1]. As previously [2], both F_{ST} and haplotype diversity statistics calculated in Arlequin were adjusted to be directly comparable to those calculated by Bayes Serial SimCoal [3]. Five million coalescent simulations were performed using Bayes Serial SimCoal, drawing parameter values N_D, P, M_E and M_L from their prior distributions: $N_D \sim U(1,1000)$, $P \sim U(0,1)$ and both M_E and $M_L \sim U(0,0.01)$. 240bp-long sequences were simulated assuming a mutation rate of 45% per million years, i.e. 2.7x10-6 per site per generation. For the modern Near Eastern and Anatolian effective female population size N_{NE} we use the same value of 1,007,170 from Bollongino *et al.* [2]. For the equivalent value in modern Europe N_E we used 7,942,392, calculated by the same methodology: multiply the summed census estimate for Europe from FAOSTAT (http://faostat.fao.org/site/291/default.aspx) (98,540,840) by an archaeological estimate for the proportion of cows in herds (0.806) [4], and then divide this by 10. Parameter values were estimated using approximate Bayesian computation (ABC) with regression adjustment as described in [5]. The same 32 summary statistics, s_i, were calculated on each simulated dataset as on the observed data, s, and a normalized Euclidean distance, $||s_i - s||$ was calculated. Simulated parameter sets were accepted if $||s_i - s|| < \delta$, where δ is implicitly calculated by setting a tolerance proportion $F_\delta = 0.1\%$, i.e. we retain the best-fitting 5,000 parameter sets. The retained parameter sets form the approximate joint posterior distribution, from which joint/marginal posterior distributions were obtained for the parameters.

Given the joint modal estimate of N_D and P (81 and 0.73, respectively; see main text), we calculated the effective population size of female cattle N_P entering Europe from the Near East at 6,400 BCE. First we calculated the effective size of the Near Eastern population N_{1400} at 1,400 generations ago (8,400 years BP), given $N_D = 80.817$ and $N_{NE} = 1{,}007{,}170$:

$$N_{1400} = N_D * \exp((\log(N_{NE}/N_D)/1750)*350) = 532.874$$

Then we multiplied this by proportion P to get the required value:

$$N_P = PN_{1400} = 0.730 * 532.874 = \mathbf{389.136}$$

In order to test the sensitivity of our analyses to the assumed values of modern effective population size in both the Near East and Europe, we ran two additional sets of simulations, identical except for using values for N_{NE} and N_E one order of magnitude greater (N_{NE} = 10,071,700, N_{NE} = 79,423,920) and one order of magnitude smaller (N_{NE} = 100717, N_{NE} = 794239). As in Bollongino *et al.* [2], we find that our estimates of N_D and P do not differ greatly from the main results and in a similar fashion. For the 10x larger model, our joint modal estimate for (N_D, P) was (69, 0.668), and for the 10x smaller model, the modal estimate was (138, 0.838). In both cases we still find the same result for the clear difference in migration rate between the Near Eastern and European populations pre- and post-5000 years BCE, essentially dropping to zero in the later stage.

Literature

1. Excoffier L, Lischer HE: **Arlequin suite ver 3.5: a new series of programs to perform population genetics analyses under Linux and Windows**. *Mol Ecol Resour* 2010, **10**(3):564-567.
2. Bollongino R, Burger J, Powell A, Mashkour M, Vigne JD, Thomas MG: **Modern Taurine Cattle descended from small number of Near-Eastern founders**. *Molecular Biology and Evolution* 2012:doi:10.1093/molbev/mss1092.
3. Anderson CN, Ramakrishnan U, Chan YL, Hadly EA: **Serial SimCoal: a population genetics model for data from multiple populations and points in time**. *Bioinformatics* 2005, **21**(8):1733-1734.
4. Vigne JD: **Les bovins (Bos taurus).** In: *Shillourokambos Un établissement néolithique pré-céramique à Chypre Les fouilles du Secteur 1* Edited by J G, Briois F, Vigne JD, Errance/ Ecole Française d' Athènes edn. Paris: Editions Errance/ Ecole Française d' Athènes; 2011: 1059-1073.
5. Beaumont MA, Zhang W, Balding DJ: **Approximate Bayesian computation in population genetics**. *Genetics* 2002, **162**(4):2025-2035.
