# Supplementary material for: The genetic prehistory of domesticated cattle from their origin to the spread across Europe
Source: BMC Genet. 2015 May 28;16:54. doi: 10.1186/s12863-015-0203-2 (PMC4445560; doi:10.1186/s12863-015-0203-2)
Supplement: Additional file 6: — Validation of ancient DNA data. Supplemental information on ancient DNA validity criteria (such as blank controls and contamination rate estimation), and explicit discussion of not fully replicated sequences. [file 12863_2015_203_MOESM6_ESM.pdf]

# Additional file 6

## Validation of ancient DNA data

None of the more than 500 hydroxyapatite, extraction and PCR blank controls yielded amplifiable amounts of bovine DNA. Significant contamination due to the presence of exogenous DNA in the lab or in the reagents can, therefore, be ruled out. Additionally, none of the approximately 3,000 PCR products sequenced by capillary electrophoresis showed evidence for bovine contamination. Specimens belonging to a very distinct bovine haplogroup called “P”, deviating from the T/Q haplogroups in at least ten positions all across the HVSI [1], were analysed in parallel during nearly every PCR (data not shown). None of the specimens presented here showed single sequences belonging to haplogroup P, and only two single sequences possibly belonging to T/Q occurred among the specimens of haplogroup P. This cross contamination rate of roughly 0.07% is in the range of pipetting errors and negligible.

For a few samples, the above mentioned strict rules for constructing consensus sequences could not entirely be fulfilled for every single amplicon, leading to gaps at the respective positions. Because of 100% sequence identity, minor parts of the HVSI sequence of specimen Tra12 were included into the analyses although they were only covered twofold.

Specimen Sam13 shows reproducible signals for both T and C at position 16,232. Amplification was performed eleven times with different primer sets and from three independently performed DNA extractions. Besides BosU5/L5 and the combination of BosU5 with BosL6 (see Table 1), primers BosH16184 TACCATGCCGCGTGAAACCA and BosL16313 TCCATCGAGATGTCTTATTTAAGAGGA [2] were used to exclude primer specific artefacts. Nine of the resulting sequences revealed both T and C alleles, two of them were retrieved using UNG during PCR. Two more sequences revealed the T allele only. There are two basic explanations for this phenomenon. The bone could be heavily contaminated by exogenous DNA that entered prior to DNA extraction. However, DNA from the only specimen that has ever been examined in the Mainz laboratory showing a polymorphism at position 16,232 was amplified more than a year earlier (Cob10). Because of our effective methods for contamination prevention described above and the overall negligible contamination rate of 0.07%, heteroplasmy is a conceivable explanation. Heteroplasmies have previously been reported in ancient DNA studies [3-5]. Moreover, they have already been proven to occur with high frequency in humans (6%), with the highest frequency (64%) in the HVSI region [6]. However, as we cannot entirely exclude contamination, this sequence was excluded from the summary statistical analyses.

To conclude, none of the hydroxyapatite, extraction and PCR blank controls contained bovine DNA. Additionally, every haplotype except Sam13 could be unambiguously determined by applying the criteria mentioned above and with no evidence of cross-contamination. Therefore, we carried out sufficient checks to present indigenous and highly reliable ancient DNA for 113 samples.

**Literature**


1. Achilli A, Bonfiglio S, Olivieri A, Malusa A, Pala M, Hooshiar Kashani B, Perego UA, Ajmone-Marsan P, Liotta L, Semino O *et al*: **The multifaceted origin of taurine cattle reflected by the mitochondrial genome**. *PLoS ONE* 2009, **4**(6):e5753.
2. Bollongino R, Edwards CJ, Alt KW, Burger J, Bradley DG: **Early history of European domestic cattle as revealed by ancient DNA**. *Biology Letters* 2006, **2**(1):155-159.
3. Rogaev EI, Moliaka YK, Malyarchuk BA, Kondrashov FA, Derenko MV, Chumakov I, Grigorenko AP: **Complete mitochondrial genome and phylogeny of Pleistocene mammoth Mammuthus primigenius**. *PLoS Biology* 2006, **4**(3):e73.
4. Gilbert MT, Drautz DI, Lesk AM, Ho SY, Qi J, Ratan A, Hsu CH, Sher A, Dalen L, Gotherstrom A *et al*: **Intraspecific phylogenetic analysis of Siberian woolly mammoths using complete mitochondrial genomes**. *Proc Natl Acad Sci U S A* 2008, **105**(24):8327-8332.
5. Edwards CJ, Magee DA, Park SD, McGettigan PA, Lohan AJ, Murphy A, Finlay EK, Shapiro B, Chamberlain AT, Richards MB *et al*: **A complete mitochondrial genome sequence from a mesolithic wild aurochs (Bos primigenius)**. *PLoS ONE* 2010, **5**(2):e9255.
6. Irwin JA, J.L. Saunier, H. Niederstätter, K.M. Strouss, K.A. Sturk, T.M. Diegoli, A. Brandstätter, W. Parson, Parsons TJ: **Investigation of heteroplasmy in the human mitochondrial DNA control region: a synthesis of observations from more than 5000 global population samples.** *J Mol Evol* 2009, **68**:516-527.
