# Supplementary figures and images for: The genetic prehistory of domesticated cattle from their origin to the spread across Europe
Source: BMC Genet. 2015 May 28;16:54. doi: 10.1186/s12863-015-0203-2 (PMC4445560; doi:10.1186/s12863-015-0203-2)

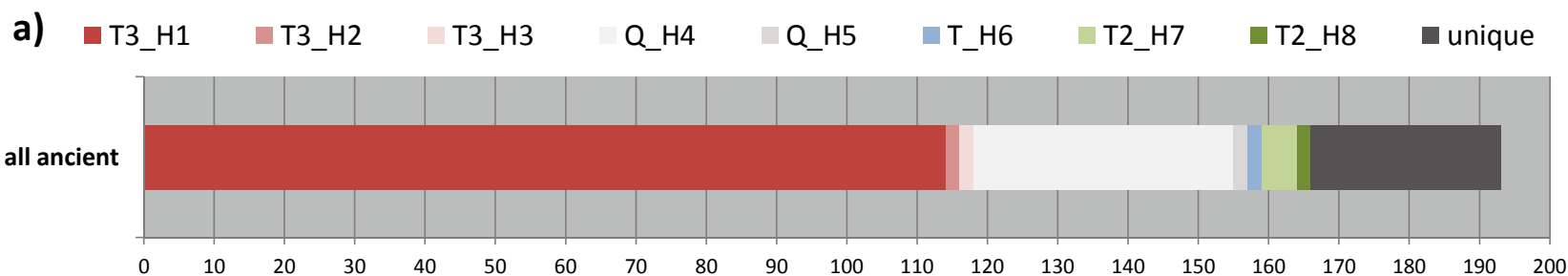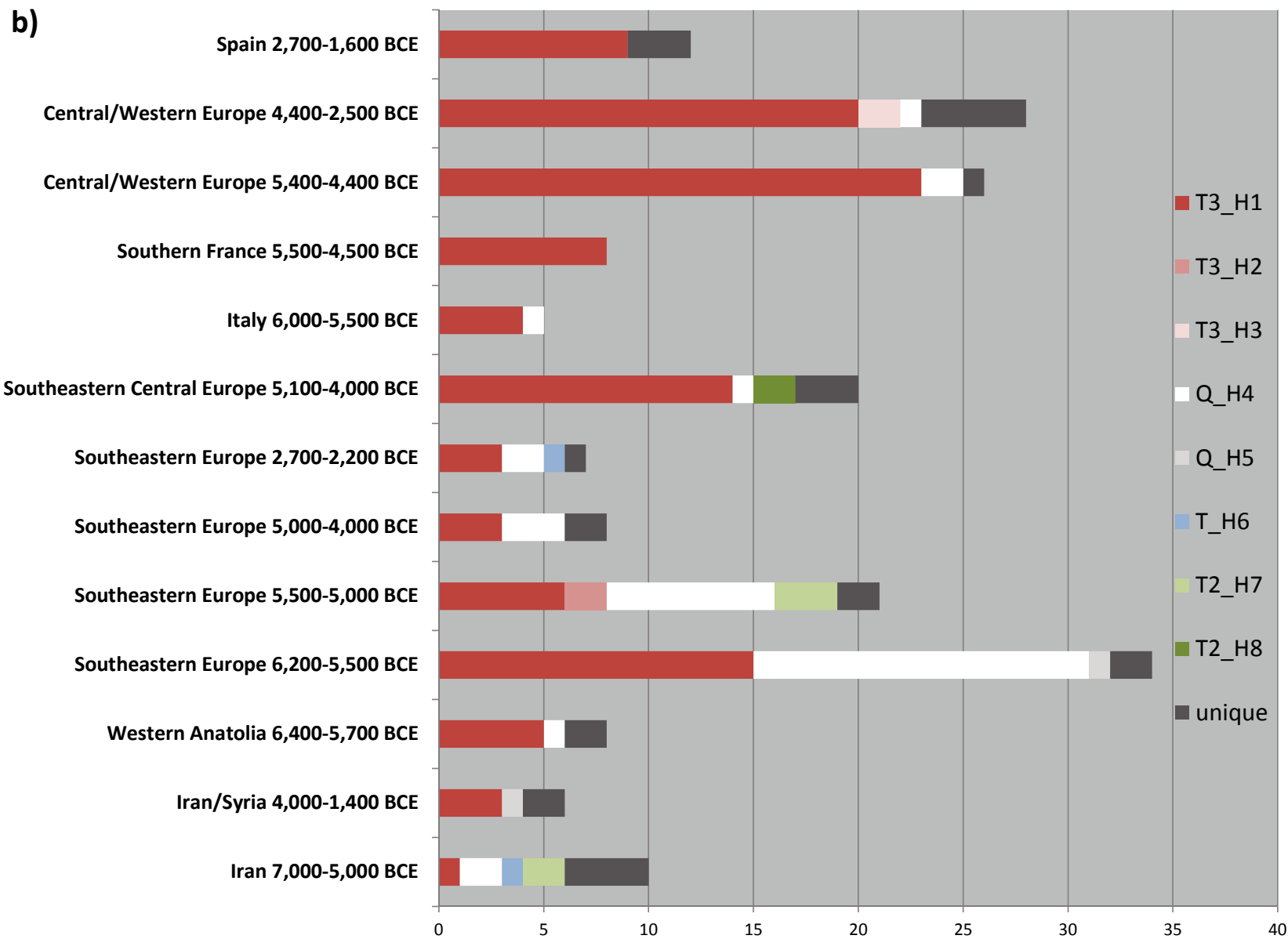

Supplement: Additional file 7: — Shared haplotypes. Shared haplotypes (H) are named and coloured according to their haplogroup and numbered consecutively. Shades of red: T3; White/light gray: Q; Shades of blue: T, T5 or T1’2’3; Shades of green: T2 (haplogroup definition according to [41]). The x-axis gives the number of sequences. a) Haplotype distribution across all 193 ancient mtDNA sequences; b) Haplotype distribution across 13 spatiotemporal groups defined by region of origin and age in BCE to the left of each bar. [file 12863_2015_203_MOESM7_ESM.pdf]
